# Asthma Endotypes and an Overview of Targeted Therapy for Asthma

**DOI:** 10.3389/fmed.2017.00158

**Published:** 2017-09-26

**Authors:** Sarah Svenningsen, Parameswaran Nair

**Affiliations:** ^1^Department of Medicine, McMaster University, Hamilton, ON, Canada; ^2^St Joseph’s Healthcare, Hamilton, ON, Canada

**Keywords:** endotype, severe asthma, omics, sputum cytometry, type 2-low asthma, type 2-high asthma

## Abstract

Guidelines for the management of severe asthma do not emphasize the measurement of the inflammatory component of airway disease to indicate appropriate treatments or to monitor response to treatment. Inflammation is a central component of asthma and contributes to symptoms, physiological, and structural abnormalities. It can be assessed by a number of endotyping strategies based on “omics” technology such as proteomics, transcriptomics, and metabolomics. It can also be assessed using simple cellular responses by quantitative cytometry in sputum. Bronchitis may be eosinophilic, neutrophilic, mixed-granulocytic, or paucigranulocytic (eosinophils and neutrophils not elevated). Eosinophilic bronchitis is usually a Type 2 (T2)-driven process and therefore a sputum eosinophilia of greater than 3% usually indicates a response to treatment with corticosteroids or novel therapies directed against T2 cytokines such as IL-4, IL-5, and IL-13. Neutrophilic bronchitis represents a non-T2-driven disease, which is generally a predictor of response to antibiotics and may be a predictor to therapies targeted at pathways that lead to neutrophil recruitment such as TNF, IL-1, IL-6, IL-8, IL-23, and IL-17. Paucigranulocytic disease may not warrant anti-inflammatory therapy. These patients, whose symptoms may be driven largely by airway hyper-responsiveness may benefit from smooth muscle-directed therapies such as bronchial thermoplasty or mast-cell directed therapies. This review will briefly summarize the current knowledge regarding “omics-based signatures” and cellular endotyping of severe asthma and give an overview of segmentation of asthma therapeutics guided by the endotype.

## Introduction

The definition of asthma has not changed in over 50 years (1). The variability in airflow that characterizes the disease may be driven by airway hyper-responsiveness or by airway inflammation that is one of the determinants of airway hyper-responsiveness (2). Despite this heterogeneity, guideline-based therapy with inhaled beta agonists and corticosteroids do not consider measurements of these individual components in routine clinical care. While the majority of asthmatics are responsive to guideline-based therapy and have reduced symptoms, improved quality-of-life, increased lung function as well as decreased exacerbation frequency (3), in approximately 5–10% of asthmatics, anticipated outcomes are not achieved (4). These patients with severe disease are responsible for the majority of indirect and direct asthma-related costs and economic burden. The advent of biologic therapies calls for a new paradigm of personalized medicine based on inflammatory endotype to better-inform who is most likely to benefit from specific targeted therapies.

Traditional asthma phenotyping (description of observable characteristics that do not provide an insight into the underlying pathobiology) classifies patients according to clinical characteristics such as exacerbating factors (allergens, exercise, and infections), age of onset, concomitant comorbidities (sinusitis and obesity), and response to treatment. More recently, unbiased clustering algorithms that have the capacity to incorporate a range of clinical variables (e.g., FEV_1_, BMI, ACQ, atopic status, and blood eosinophils) have been applied to large patient datasets to objectively identify clinical phenotypes of asthma. Such datasets include the Severe Asthma Research Program (SARP) (5), Airways Disease Endotyping for Personalized Therapeutics (ADEPT) (6), and Unbiased Biomarkers for the Prediction of Respiratory Disease Outcomes (U-BIOPRED) (6, 7) cohorts, which have been partitioned into up-to five clinical phenotypes. However, composites of observable characteristics do not provide us insight into mechanisms of persistent symptoms, physiological abnormalities, or inflammation, and therefore are of limited value to choose the appropriate biological agent. As opposed to phenotypes, characterizing severe asthmatics according to their endotype, a term applied to a “subtype of a condition that is defined by a distinct functional or pathophysiological mechanism,” may be more useful to directing therapy (8). This notion is strongly supported by the efficacy of biologic therapies that target-specific inflammatory mediators (e.g., IL-5) in well-defined patients characterized based on inflammatory biomarkers (9, 10). Currently, however, asthma management guidelines fail to adequately emphasize the measurement of the inflammatory component of airway disease (bronchitis).

Inflammatory endotype characterization should be considered a central component of the workup and management of severe asthma. Widespread acceptance of this notion, however, has been slow, perhaps because there is no consensus as to how to best identify asthma endotypes and what therapy to use for a given endotype. While novel omics-based signatures of severe asthma have emerged, they have not been evaluated clinically. We suggest that asthma endotype characterization can be reliably done on the nature of underlying airway inflammation assessed by sputum cytometry (1). This review aimed to summarize the current knowledge regarding cellular endotyping and novel “omics-based signatures” of severe asthma and give an overview of segmentation of asthma therapeutics guided by the inflammatory endotype. Molecular pathways and mechanisms associated with asthma endotypes were recently reviewed and therefore have not been discussed in detail here.

## Inflammatory Endotypes

Wenzel et al. (11) defined two distinct inflammatory endotypes of severe, corticosteroid-dependent asthma based on the presence or absence of eosinophils in endobronchial biopsy and lavage. Since this landmark study, T-helper type 2 (Th2)-high and Th2-low have remained the most well recognized and described endotypes of severe asthma. The Th2-high endotype is characterized by the presence of eosinophilic airway inflammation, whereas the Th2-low endotype is usually characterized by neutrophilic or paucigranulocytic airway inflammation. While several non-invasive biomarkers exist for the detection of the Th2-high endotype [blood eosinophils, serum IgE, serum periostin, and exhaled nitric oxide (eNO)], sputum cytometry is currently the most clinically validated quantitative and responsive method to assess airway inflammation. Perceived difficulties in implementing this approach in routine clinical practice have limited its widespread use. In fact, as described by Lim and Nair (12), many easily accessible biomarkers with demonstrated clinical utility remain confined to the research arena or are only exploited in a small number of specialized academic centers. This may be due to the lack of measurement standardization, validated diagnostic or predictive thresholds, and evidence-based reference guidelines to inform how biomarkers should be used and interpreted in clinical practice. Cost and infrastructure constraints also limit generalizability of validated biomarkers. To overcome these issues, readily available or point-of-care diagnostic methods are welcomed. Quantitative cytometry of induced or spontaneous sputum is currently the most sensitive and specific non-invasive method to directly characterize the type and severity of airway inflammation in asthma (13). Importantly, standardized methodology for sputum induction, processing, and quantification (14, 15) is safe (16) and well tolerated by the majority of patients (17–19). The nature of bronchitis assessed by sputum cytometry can be stratified into four groups based on the percentage of sputum granulocytes: (1) eosinophilic, (2) neutrophilic, (3) mixed-granulocytic (eosinophils and neutrophils elevated), and (4) paucigranulocytic (eosinophils nor neutrophils elevated) (17, 20). In non-smoking healthy adults, Belda et al. established the 90th percentile for sputum eosinophil and neutrophil counts, reporting 1.1 and 64.4%, respectively, with a total cell count of 9.7 million cells/g (21). However, standardized stratification cutoffs have not been established in asthma and as a result have varied between studies. Proposed thresholds for sputum eosinophilia have ranged between >1.1 and 4% of the total cell count (17, 20, 22) with studies strongly suggesting that a cutoff of >3% is clinically relevant and can be used to guide treatment and reduce exacerbations (23). To indicate neutrophilia, thresholds >61% of the total cell count have been proposed (20). Overlooked by many centers, it is important to acknowledge that the presence of eosinophil free granules also indicates uncontrolled eosinophilic bronchitis (24). The prevalence of these groups have been reported in stable, severe, and exacerbated disease with the proportion of eosinophilic asthma ranging from 35 to 50% (17, 20). The groups differ with respect to their clinical characteristics and response to therapy. Patients with mixed-granulocytic asthma have more severe airflow obstruction, a higher frequency of exacerbations and daily wheeze, and increased health care utilization than patients with either eosinophilic or neutrophilic bronchitis alone (25). It is also important to note that asthmatics who exhibit concordant blood and sputum eosinophilia experience more airway symptoms than those with isolated blood or sputum eosinophilia alone (19).

As mentioned above, there is no consensus on the specific definition of inflammatory cellular endotypes. Based on our clinical experience of over 20 years in over 5,000 patients, a patient can be determined to have eosinophilic asthma if there is evidence of elevated sputum (>3% with or without degranulation) and/or blood eosinophils (≥400 cells/μL) on at least two occasions, and if treatment strategies aimed at suppressing eosinophils are effective in controlling symptoms and exacerbations (26). Likewise, a patient can be determined to have neutrophilic asthma if there is evidence of elevated neutrophils (≥64%) but not eosinophils (<3%) with an increased total cell count (≥9.7 million cells/g) on at least two occasions and if treatment strategies aimed at suppressing eosinophils are not effective in controlling symptoms and exacerbations. Patients can exhibit features of both the eosinophilic and neutrophilic endotype. A patient can be determined to have mixed-granulocytic asthma if there is evidence of both neutrophils and eosinophils, independently or concurrently, on at least two occasions. Finally, a patient can be determined to have paucigranulocytic asthma if there is no evidence of elevated eosinophils (<3%) or neutrophils (<64%) and if treatment strategies aimed at suppressing eosinophils and neutrophils are not effective in controlling symptoms and exacerbations. The workup and therapy regimen for these inflammatory endotypes have been described at the end of this review.

## Novel Omics Endotyping Strategies

The maturation of omics-based technologies has facilitated the investigation of transcriptomics (27–33), proteomics (25, 34), and metabolomics (35, 36) to better understand the molecular characteristics of asthma, which have all been recently reviewed. Large multicenter initiatives are now ongoing, including the U-BIOPRED project (37), which aim to identify distinct severe asthma endotypes by integrating inflammatory biomarkers derived from “omics” and clinical data. Thus far, omics measurements have been utilized to (1) identify asthma endotypes (38), (2) identify genes related to inflammatory characteristics (39), and (3) to describe the molecular characteristics of clinical asthma phenotypes (6). Studies are required to investigate the clinical benefit of these more sophisticated and computationally intensive endotyping strategies, both to initiate the appropriate treatment and to longitudinally monitor responses to various anti-inflammatory (including biologics) therapies in asthma.

### Transcriptomics

When compared with non-asthmatics, whole-genome expression in peripheral blood of severe asthmatics is different such that a severe asthma disease signature comprised of nearly 1,700 genes was identified by Bigler et al. (38). Within severe asthma, distinct gene signatures associated with eosinophilia, mast cells, and group 3 innate lymphoid cells have been identified in patients with adult-onset asthma when compared with patients with childhood-onset asthma (32). Beyond asthma versus non-asthma stratification, numerous studies (summarized in Table 1) have aimed to define transcriptomic endotypes of asthma by analyzing differential gene expression in bronchial epithelium (27) and induced sputum samples (28, 30, 31, 33). Woodruff and colleagues were the first to identify two evenly sized “Th2-high” and “Th2-low” subgroups of mild, steroid naive asthma based on unsupervised hierarchical clustering of the expression levels of three Th2 cytokine induced genes (POSTN, CLCA1, and SERPINB2) in bronchial epithelial brushings. These subgroups were different with respect to their expression levels of IL-5 and IL-13 within the airway, airway hyper-responsiveness, IgE, blood and airway eosinophilia, and reticular basement membrane thickness. Not surprisingly, Th2-high gene expression was predictive of corticosteroid responsiveness as clinically relevant improvements in FEV_1_ following 8 weeks of fluticasone use were only observed in the Th2-high group (27). Wilson et al. (39) identified seven genes (COX-2, ADAM-7, SLCO1A2, TMEFE2, and TRPM-1, and two unnamed genes) in bronchial brushing samples with expression levels that were moderately correlated with submucosal eosinophils, suggesting that they may also predict corticosteroid responsiveness. Given the limited clinical applicability of invasive bronchoscopic samples, Woodruff and colleagues went on to evaluate the variable expression of 14 genes relevant to Th2 inflammation in induced sputum samples (30). Generating a quantitative composite metric of IL-4, IL-5, and IL-13 gene expression, termed the “Th2 gene mean,” the study population was again dichotomized into Th2-high (70%) and Th2-low (30%) subgroups (30). When compared with the Th2-low cluster, the Th2-high cluster had higher eNO levels as well as sputum and blood eosinophil counts (30). It is notable that sputum (AUC = 0.89) and peripheral blood (AUC = 0.89) eosinophil counts alone, but not eNO (AUC = 0.76), performed well as biomarkers of Th2-high asthma as assessed by the sputum cell “Th2 gene mean” (30). Acknowledging the limitations of analyzing pre-selected genes, Baines et al. (28) subjected whole-genome gene expression profiles from induced sputum of adults with stable asthma to unsupervised hierarchical cluster analysis. Three distinct transcriptional asthma phenotypes (TAPs) were identified that had similarities to previously defined sputum inflammatory phenotypes of eosinophilic (TAP1), neutrophilic (TAP2), and paucigranulocytic (TAP3) asthma (28). In fact, 92% of the differentially expressed genes between the TAPs overlapped when the population was grouped according to sputum quantitative cell count (eosinophilic, neutrophilic, and paucigranulocytic). The same investigators subsequently identified a sputum gene expression signature comprised of six genes (CLC, CPA3, DNASE1L3, IL1B, ALPL, and CXCR2) that was able to discriminate asthmatics according to their inflammatory endotype and predict ICS response (29). Of most interest, the six-gene expression signature outperformed the ability of sputum eosinophil count to predict corticosteroid response (FEV_1_ responder vs. non-responder; AUC = 91.5) (29). Somewhat contradictory to the findings of Baines et al., when unbiased hierarchical clustering was performed on 508 genes that were differentially expressed between severe asthmatics with and without eosinophilic airway inflammation confirmed by sputum cytometry, one Th2-high and two non-Th2 transcriptome-associated clusters (TACs) were defined (33). When examining the distribution of the three TACs according to sputum inflammatory endotype, two TACs were associated with eosinophilic (TAC1 or TAC3) and neutrophilic (TAC2 or TAC3) inflammation, suggesting that two distinct transcriptional signatures are associated with both sputum eosinophilia and neutrophilia (33). Similarly, Yan et al. (31) did not detect significant between cluster differences in sputum cell differentials suggesting an imperfect association with Th2 pathophysiology. Taken together, conflicting evidence surrounds the association between transcriptomic endotypes and sputum quantitative cell count.

**Table 1:** Summary of transcriptomics studies.

Reference	Subjects	Transcriptomic analysis	Computational analysis		Predictive investigation?
			Approach	Result	
**Blood**					
Bigler et al. (38)	Severe asthma non-smokers (*n* = 309); severe asthma current/ex-smokers (*n* = 110); mild–moderate asthma (*n* = 87); healthy controls (*n* = 100)	Microarray	Whole-genome gene expression data were filtered and 1,693 entities differentially expressed between severe asthmatics and non-asthmatics (“severe asthma disease signature”) were subjected to unsupervised hierarchical clustering and topological analysis	Two clusters: “Severe asthma-enriched cluster” and “mixed cluster”	No

**Bronchial brushings**					
Woodruff et al. (27)	Mild-to-moderate asthma (*n* = 42); healthy controls (*n* = 28)	Microarray and qPCR	Unsupervised hierarchical clustering based on the gene expression profile of three IL-13-inducible genes (POSTN, CLCA1, and SERPINB2)	Two clusters: “Th2-high” and “Th2-low” asthma	Yes, ICS response
Wilson et al. (39)	Severe asthma non-smokers (*n* = 46); severe asthma current/ex-smokers (*n* = 16); mild–moderate asthma (*n* = 34); healthy controls (*n* = 41)	Microarray	No cluster analysis. Association of gene expression profiles with eosinophil and neutrophil counts evaluated using a general linear model	NA	No

**Sputum**					
Baines et al. (28, 29)	Adults with stable asthma (*n* = 59); healthy controls (*n* = 13)	qPCR	Whole-genome gene expression data (22,218 entities) were filtered and 7,436 entities present in all asthmatics were subjected to unsupervised hierarchical clustering	Three “transcriptional asthma phenotypes”	Yes, ICS response
Peters et al. (30)	Asthma (*n* = 37); healthy controls (*n* = 15)	qPCR	Supervised analysis of gene expression profiles of 14 genes relevant to Th2 inflammation	Single quantitative metric: “Th2 gene mean”	Yes, Th2-high and Th2-low endotype
Yan et al. (31)	Asthma (*n* = 100); healthy controls (*n* = 12)	Microarray	5,500 gene expression profiles from 186 Kyoto Encyclopedia of Genes and Genomes pathways were used to assess the pathway-based distance between samples followed by unsupervised k-means clustering	Three “transcriptomic endotypes of asthma”	No
Kuo et al. (33)	Moderate-to-severe asthma (*n* = 104); healthy controls (*n* = 16)	Microarray	508 differentially expressed genes between eosinophil (≥1.5%) and non-eosinophil (<1.5%) associated sputum inflammation were subjected to unbiased hierarchical clustering	Three “transcriptome-associated clusters”	No

**Sputum, nasal brushings, bronchial brushings, and biopsies**					
Hekking et al. (32)	Adults with adult-onset (*n* = 253) and childhood-onset severe asthma (*n* = 158)	Microarray	105 predefined genes associated with the presence of asthma, leukocytes, and induced lung injury were subjected to gene set variation analysis to form gene signatures associated with adult-onset severe asthma	Significantly different asthma, leukocyte, and induced lung injury gene signatures in adult-onset severe asthma patients (bronchial brushings *n* = 6; nasal brushings *n* = 5; sputum *n* = 3)	No

### Proteomics

While gene expression studies have dominated the omics landscape, it is their translated products, the proteins, which mediate airway inflammation in asthma by regulating cell recruitment. Numerous studies, limited by sample size, have investigated the “proteomic profile” of asthma in bronchoalveolar lavage fluid (BALF) (40, 41), bronchial biopsy (42), and sputum supernatants (43, 44). One relatively large study by SARP investigators (34) focused on 18 cytokines detectable in BALF and discriminated mild-to-moderate and severe asthmatics into four groups based on cytokine expression. The groups were independent of corticosteroid use and phenotypically distinct with respect to BALF cellularity and lung function. In another study, Hastie et al. (25) investigated the hypothesis that sputum inflammatory granulocytes define phenotypic subgroups of asthma with different patterns of inflammatory proteins. Protein microarray data of induced sputum were stratified by sputum cell differential (eosinophilic: ≥2% eos and <40% neuts; neutrophilic: <2% eos and ≥40% neuts; mixed-granulocytic: ≥2% eos and ≥40% neuts; paucigranulocytic: <2% eos and <40% neuts) and revealed differentially increased inflammatory proteins between the groups (25). Of note, 19 inflammatory mediators were significantly elevated in those asthmatics with neutrophilic bronchitis, a subset of which were correlated with neutrophil counts.

### Metabolomics

Metabolomics, the exploration of biochemical molecules derived from metabolic processes, was recently reviewed for its applications in asthma (36). Studies to date strongly suggest that metabolic profiles measured in exhaled breath, urine, plasma, and serum may be applied as a point-of-care tool to discriminate asthma endotypes (36). Of most interest, “breathomics” (45) profiles volatile organic compounds in exhaled breath using an electronic nose and has demonstrated ability to discriminate asthmatics from healthy controls (46–48). Electronic nose “breathprints” are correlated with the percentage of sputum eosinophils (48, 49). One highly relevant study concluded that exhaled breath analysis outperformed the ability of both eNO and the percentage of sputum eosinophils to predict corticosteroid response in asthmatics from whom corticosteroids had been withdrawn (48).

### Important Considerations and Future Studies

There are numerous limitations to clustering techniques applied in the studies discussed above; therefore, these results should be interpreted with caution. One such concern is cluster stability. Specifically, clusters identified at a specific point in time may not be reproducible at subsequent time points. Longitudinal stability assessment of the clusters that have been described is required to understand how they behave over time and in response to treatment and/or exacerbations. Similarly, different clusters may be identified across different asthma populations and therefore validation of cluster classification in independent cohorts of asthmatic patients is necessary to understand generalizability. These important considerations were evaluated for the first time by Loza et al. (6) who defined four severe asthma phenotypes in two independent severe asthma cohorts (ADEPT and U-BIOPRED), performing both external validation and longitudinal stability assessments. Cluster number and composition may also be influenced by the clustering methodology applied. Finally, it is also important to consider statistical robustness as the majority of studies to date define clusters comprised of a small number of asthma patients and hence have limited statistical power.

None of the omics-signatures discussed above have been translated to clinical practice as prognostic or predictive tests. In fact, the clinical utility of these signatures is currently unknown as the majority of studies to date have been observational and hypothesis generating. The ultimate potential of a biomarker-based clinical test is dependent on its analytical and clinical validity in addition to its clinical utility. A 30-point checklist of criteria, developed by McShane et al., should be considered to gauge the potential clinical utility of the omics-based signatures discussed in this review (50). Important considerations include those related to the specimen and assays used, and the appropriateness of the statistical methods used to develop and validate the signature (50). Studies are now required to address the paucity of evidence concerning (1) the longitudinal variability of omics data and endotypes and (2) the responsiveness of omics to therapies. Randomized clinical trials (RCTs) will be necessary to definitively confirm the clinical utility of novel omics-based signatures and design consideration for biomarker RCTs have been proposed (51). Specifically, intervention studies will be necessary to shed light on the capacity of these signatures to direct personalized therapy and to adjust doses of drugs during exacerbations in severe asthma. The qualitative nature of omics is an obvious limitation, therefore the development and application of qualitative metrics are certainly desirable. Similar to that described by Hinks et al. (52), multi-dimensional clinicopathobiologic clustering should also be considered to maximally leverage the measurements available to tertiary care clinicians. It is clear however that omics-based endotypes have similar molecular, physiological, and clinical characteristics to the inflammatory endotypes of eosinophilic, neutrophilic, mixed-granulocytic, and paucigranulocytic asthma.

## Inflammatory Endotype-Guided Therapeutic Strategy

**Figure 1:**
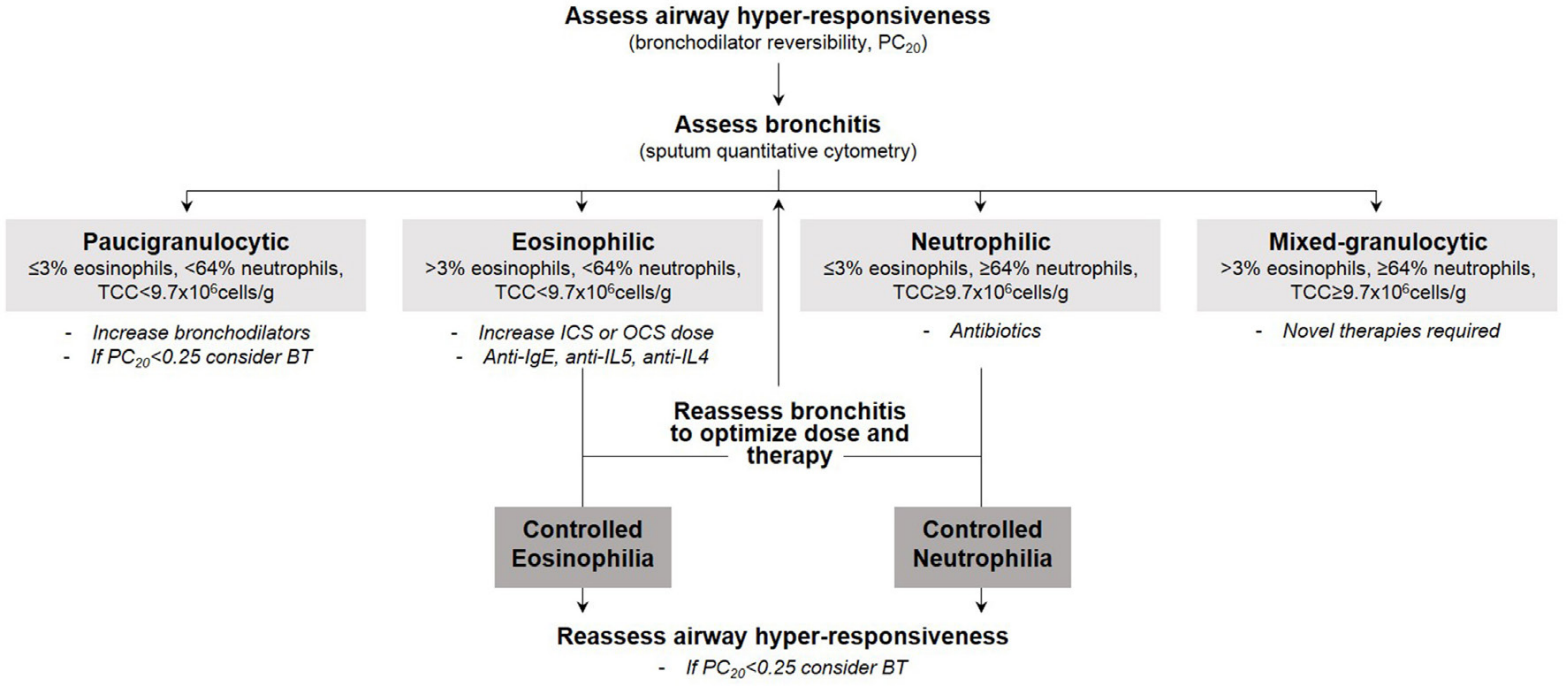
Our therapeutic strategy in severe asthma guided by inflammatory endotype and severity of airway hyper-responsiveness.

In this section, we focus our discussion on the segmentation of therapy in severe asthma [as defined by the European Respiratory Society/American Thoracic Society (4)] guided by inflammatory endotype. We support the notion that the specific nature of bronchitis reveals the underlying mechanism driving the bronchitis and therefore predicts therapy response. As summarized in Figure 1, our therapeutic strategy is dependent on the type of bronchitis (assessed using quantitative cytometry in induced sputum) (53) and severity of airway hyper-responsiveness (assessed using methacholine inhalation challenge). We emphasize the importance of identifying the particular component of airways disease that drives symptoms in each patient prior to therapy selection. Furthermore, the components of airways disease should be assessed and subsequently reassessed to optimize therapy such that symptom and inflammation control is achieved. Careful endotyping is probably not necessary to manage most patients with asthma. This strategy has not been shown to reduce exacerbations in patients with mild asthma (reference our own Jayaram et al., study). It is currently recommended only for patients with more severe asthma.

### Management of the T2-High Endotype

Although most commonly referred to as Th2-high, type 2 (T2)-high has emerged as a more appropriate and inclusive term given the involvement of numerous cell types (including type 2 innate lymphoid cells and natural killer cells) that are outside the originally described Th2 cell population (54). *Eosinophilic bronchitis* indicates a T2-driven mechanism and is usually steroid responsive. When bronchitis is eosinophilic in nature with a differential cell count of more than 3% (or eosinophil-free granules are observed), inhaled corticosteroids should be initiated and the dose titrated to keep the sputum eosinophil count below 3%. In situations where high-dose inhaled corticosteroids do not control sputum eosinophilia, oral corticosteroids are required. In corticosteroid-treated patients, absent eosinophils may suggest that the current steroid dose is unwarranted and therefore should be reduced to avoid over-treatment. In RCTs in adults, moderate-to-severe asthmatics managed by normalizing induced sputum eosinophils had significantly reduced exacerbations and hospital admissions when compared with those managed by national asthma guidelines (23, 55). When this strategy was applied clinically in 52 OCS-dependent asthmatics, maintained symptom control, reduced exacerbations, and preserved lung function was observed over 5 years (56). Novel biologic therapies should be considered for their steroid sparing effect, also for the minority of patients who are corticosteroid-insensitive (unresolved sputum eosinophilia despite high doses of oral corticosteroids) (57).

**Figure 2:**
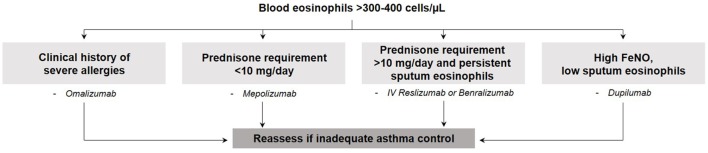
General scheme to choose the appropriate monoclonal antibody based on simple clinical features.

To date, all biologic therapies that have been approved and the majority of those in development aim to target T2 inflammation [recently reviewed (58)] and are therefore directed toward the T2-high eosinophilic asthma endotype. Detailed illustrations of asthma pathobiology and the mechanism of action of targeted therapies are provided in recent reviews (59, 60). Anti-IgE [omalizumab (61)] therapy was the first approved monoclonal antibody and is effective in patients with allergic asthma, confirmed by a positive skin prick test and serum IgE levels ≥30 IU/mL. Two anti-IL-5 therapies are approved [mepolizumab (62) and reslizumab (63)] and one is in phase 3 development [benralizumab (64, 65)] for use in severe eosinophilic asthma. With the potential to block both IL-4 and IL-13, one anti-IL-4 receptor alpha therapy is currently in phase 3 development (dupilumab) following successful phase 2b trials (66, 67). The efficacy of strategies targeting IL-13 alone [lebrikizumab (68) and tralokinumab (69)] is inconclusive as only modest clinical benefit has been shown. Drugs that target both IL-4 and IL-13 signaling (e.g., dupilumab) have reported more clinically relevant effects in phase 2 clinical programs. The reason for this difference is not immediately obvious. Perhaps one of the reasons may be related to the lack of accurate endotyping to identify patients in whom IL-13-mediated biology was not the dominant pathobiology of asthma. Selecting patients with significant airway hyper-responsiveness and mucus secretion may have demonstrated greater clinical effect. Other novel therapies that are currently being investigated include anti-thymic stromal lymphopoietin (70), IL-33 blocking agents (71), and prostaglandin antagonists (CRTH2) (72). A general scheme to choose the appropriate monoclonal antibody based on simple clinical features (e.g., clinical history of allergy, severity of asthma based on the dose of corticosteroids, and readily available biomarkers such as blood and sputum eosinophils and fraction of eNO) is shown in Figure 2. This is based on our clinical experience, the lack of evidence supporting omalizumab in prednisone-dependent asthmatics (73), the lack of efficacy of mepolizumab 100 mg subcutaneous in patients with persistent sputum eosinophilia despite high-dose inhaled and oral corticosteroid use (74), and the efficacy of benralizumab (75) and reslizumab (76) in severe prednisone-dependent patients. However, this approach needs to be prospectively validated in clinical trials.

### Management of the T2-Low Endotype

*Neutrophilic bronchitis* with a raised total cell count is suggestive of a non-T2-driven disease and is not usually steroid-responsive, but instead a predictor of response to antibiotics. Macrolides, including clarithromycin (77) and azithromycin (78), have demonstrated effectiveness in non-eosinophilic asthmatics. To identify the causative pathogen that may help to direct antibiotic therapy, molecular microbiology, and extended cultures, including 16 s deep sequencing, should be considered in those patients with recurrent infective exacerbations. Logically, neutrophilic bronchitis may be a predictor of therapies targeted at pathways that lead to neutrophil recruitment such as TNF, IL-1, IL-6, IL-8, IL-23, and IL-17. Several small molecule anti-neutrophilic biologics have been developed, although there are currently no active phase 3 trials. Such molecules include CXCR2 antagonists (79, 80), 5-lipoxygenase-activating protein inhibitors (81), anti-IL-17 (82), and anti-TNFα (83). It is evident that few therapeutic options exist for these patients; therefore, intense study of the underlying mechanisms contributing to the T2-low endotype is urgent. Similarly, despite the severity of their disease, currently there are no treatment options for patients with *mixed-granulocytic asthma*. In fact, no interventions have been evaluated for this specific inflammatory endotype although preliminary evidence suggests therapies targeting the IL-6 pathway may be beneficial (84).

Asthmatics with *paucigranulocytic bronchitis* may not warrant anti-inflammatory therapy as symptoms in these patients may be driven solely by smooth muscle dysfunction (airway hyper-responsiveness). Therefore, these patients may benefit from smooth muscle-directed therapies such as additional bronchodilators and long-acting anti-muscarinic antagonists, mast-cell directed therapies, or in the most severe cases, bronchial thermoplasty. Our clinical experience suggests that bronchial thermoplasty is indicated when severe airway hyper-responsiveness (PC_20_ < 0.25) and frequent exacerbations persist despite absent or controlled airway inflammation (85); however, clinical trials are required to confirm this hypothesis. Bronchial thermoplasty after therapy has been optimized to control eosinophilic and/or neutrophilic inflammation has been previously described by Cox et al. (85) for those patients with persistent symptoms. Although the mechanism of action is uncertain, bronchial thermoplasty aims to attenuate airway smooth muscle mass through the delivery of localized thermal energy (86).

## Concluding Remarks

One of the major challenges of respiratory medicine is the management of patients with severe asthma. Identifying a specific endotype may have profound implications on advanced targeted therapy selection and intern clinical outcomes. It should be acknowledged that the mere presence of a particular gene, protein or a cell do not necessarily make them a therapeutic target or disease biomarker. Teasing out “association” from “causality” is of paramount importance. Koch’s postulates, described in 1890, remain a useful benchmark to establish whether there is causal relationship between a particular molecular or cellular observation and a disease presentation. Persistence of a particular cell type or cytokine and their temporal association with exacerbation and resolution provide strong evidence to support a causative role. A consensus as to how to best identify asthma endotypes and what therapy to use for a given endotype is now required. In the meantime, quantitative cell counts in sputum provide the most reliable method to identify T2 (most eosinophilic) and non-T2 (most neutrophilic) inflammatory processes.

## Author Contributions

PN conceived the idea. SS prepared the first draft. PN and SS edited and reviewed the manuscript and have approved the final version of the manuscript.

## Conflict of Interest Statement

PN has received consultancy fees from AstraZeneca, Boehringer Ingelheim, Sanofi, Teva, Knopp, Theravance, and Roche; research support from GlaxoSmithKline, AstraZeneca, Sanofi, Boehringer Ingelheim, Roche, and Novartis; and lecture fees from Roche, AstraZeneca, and Novartis.
